# Road RAGE?: The Role of Diesel Particulate Matter in Lung Inflammation

**DOI:** 10.1289/ehp.119-a132a

**Published:** 2011-03

**Authors:** Kellyn S. Betts

**Affiliations:** **Kellyn S. Betts** has written about environmental contaminants, hazards, and technology for solving environmental problems for publications including *EHP* and *Environmental Science & Technology* for more than a dozen years

Diesel particulate matter (DPM) is a nearly ubiquitous environmental pollutant. It is known to be inflammatory and is linked to a plethora of health effects including asthma, chronic obstructive pulmonary disease, and pulmonary fibrosis. New research sheds light on which components of DPM are harmful to the lung and what mechanisms they trigger **[*EHP*** **119(3):332–336; Reynolds et al.].**

The authors focused on receptors for advanced glycation end-products (RAGE), cell-surface proteins expressed in many cell types. Previous research performed in the same laboratory documented that RAGE can be activated in response to cellular stress resulting from exposure to particles in cigarette smoke. The authors hypothesized that exposure to DPM generated by fuel combustion could induce RAGE in the epithelial cells lining the lungs.

The team studied effects of DPM exposure in human primary pulmonary epithelial cells and R3/1 cells, an immortalized aveolar type 1 cell line derived from rats. They found that the quantities of *RAGE* messenger RNA and protein increased by approximately 100% in both cell types after exposure to DPM for 2 hours, compared with controls. By demonstrating that RAGE is indeed upregulated following exposure to DPM, the authors identified a surface signaling mechanism involved in inflammatory responses triggered by DPM exposure.

From there, the scientists identified some of the downstream signaling effects associated with RAGE upregulation. Their gene reporter experiments showed DPM exposure caused significant translocation of nuclear factor κB (NF-κB), a potent proinflammatory mediator, into the nucleus of R3/1 cells, where it can promote the expression of more than 200 genes. Through experiments involving the inhibition of RAGE with small interfering RNA (siRNA), the team confirmed that DPM-induced NF-κB activation is mediated in part by RAGE expression.

The scientists also documented that exposure to DPM increased the synthesis and secretion of two NF-κB targets (IL-8, a chemokine, and MCP-1, a cytokine) by the R/31 cells. These molecules were secreted to a lesser extent, but were not completely inhibited, in cells transfected with siRNA for RAGE prior to DPM exposure, which suggests other factors and pathways also are involved in inflammatory responses to DPM.

The new research is also significant for contradicting conventional wisdom that only “fresh” DPM is biologically active. The work suggests that even “aged” DPM that has been suspended in the atmosphere for more than a decade is capable of biological activity, which has important public health implications given the abundance of this pollutant in the atmosphere.

## Figures and Tables

**Figure:**
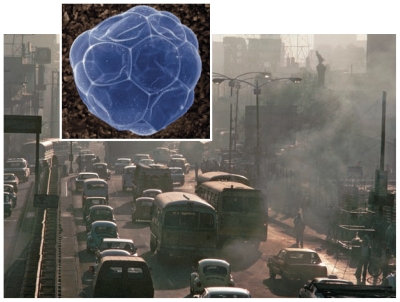
Diesel particulate matter created by vehicular traffic has been linked to a plethora of adverse pulmonary and cardiovascular effects.

